# Determination of Diniconazole in Agricultural Samples by Sol-Gel Immunoaffinity Extraction Procedure Coupled with HPLC and ELISA

**DOI:** 10.1371/journal.pone.0046929

**Published:** 2012-10-08

**Authors:** Zhenjiang Liu, Yahui Jin, Minghua Wang

**Affiliations:** Department of Pesticide Science, College of Plant Protection, Nanjing Agricultural University, Jiangsu Key Laboratory of Pesticide Science, Key Laboratory of Integrated Management of Crop Diseases and Pests, Ministry of Education, Nanjing, China; New York University, United States of America

## Abstract

**Background:**

In the European Union (EU), the use of diniconazole-M is no longer authorized. However, residues of diniconazole-M occur in various plant commodities.

**Methodology/Principal Findings:**

A selective and simple analytical method for the trace level determination of diniconazole in soil, fruit, vegetables and water samples was developed based on immunoaffinity extraction followed by Enzyme-linked immunosorbent assay (ELISA) and the high-performance liquid chromatography (HPLC) analysis. The ELISA was based on monoclonal antibodies highly specific to diniconazole and was a fast, cost-effective, and selective screening method for the detection of diniconazole. The results of the ELISA correlated well with gas chromatography (GC) results, with the correlation coefficient of 0.9879 (n = 19). A simple gel permeation chromato- graphy clean-up method was developed to purify extracts from matrices containing high amounts of fat and natural pigments, without the need for a large dilution of the sample. The immunoaffinity column (IAC) capacity was 0.180 mg g^−1^. The columns could be re-used approximately 20 times with no significant alteration in capacity. The recoveries from complex samples were in the range of 89.2% to 96.1% with a relative standard deviation (RSD) of 0.770%–6.11% by ELISA. The results were in good agreement with those obtained by HPLC method.

**Conclusion/Significance:**

The IAC extraction procedure coupled with HPLC and ELISA analysis could be also used as alternative effective analytical methods for the determination of diniconazole concentrations in complex samples.

## Introduction

Diniconazole [(E)-(RS)-1-(2,4-dichlorophenyl)-4,4-dimethyl-2-(1H-1,2,4-triazol-1-yl) -pent-1-en-3-ol] belongs to the group of triazole fungicides. It has a systemic action, travelling in the plant by apoplastic pathways, and acts via ergosterol biosynthesis inhibition [1]. It is widely used to control a broad range of fungal diseases in many crops.

In the European Union (EU), the use of diniconazole-M (E, R-diniconazole) is no longer authorized. But residues of diniconazole-M occur in various plant commodities. Diniconazole-M was found to be the principal component of the residue in foliage and present at significant levels in grain. A risk assessment is in principle not required considering that the use is no longer authorized in the EU, but the default maximum residue limit (MRL) of 0.01 mg kg^−1^, as defined by Regulation (EC) No 396/2005, provides a satisfactory level of protection for the European consumer [1].

Many methods for the determination of diniconazole residues in different types of samples have been reported, these methods include gas chromatography-electron capture detector (GC-ECD) [2]–[6], gas chromatography-mass spectrometry [2], [7], high-performance liquid chromatography-ultraviolet detection (HPLC-UV) [8]–[11] and high-performance liquid chromatography-mass spectrometry [12]. However, current analytical methods are costly and time-consuming. Therefore, there is a growing demand for more rapid and economical methods for determining pesticide residues. Enzyme-linked immunosorbent assay (ELISA) is the basis of fast, sensitive, cost-effective, and selective method for the detection of pesticide residues. An ELISA based on a polyclonal antibody against diniconazole was first developed by Jiang et al., which used large volumes of organic solvents and a multi-step extraction procedure from complex samples purification [13].

Classic sample purification methods include liquid-liquid partitioning and solid-phase extraction (SPE). SPE is actually the technique of choice for sample preconcentration and cleanup. However, the reversed phase sorbents commonly used (C18 and polymeric phases) are non-selective. Significant amounts of other matrix components co-extract during the SPE of more complex samples, and may severely interfere in the analysis of target analytes [14]. Although selectivity is less of a problem when mass spectrometry is coupled to HPLC, co-extracted material can affect the detector response or decrease the capacity of the SPE pre-column to quantitatively retain the analytes [15]. Immunoaffinity column (IAC) is a selective purification column with- out co-extracted material, allowing the isolation and enrichment of target analytes from complex sample matrices.

In this paper, a sensitive ELISA based on monoclonal antibodies for the detection of diniconazole residues in water and complex samples was described. The ELISA performance was evaluated by GC using spiked samples. The employment of a sol-gel-entrapped monoclonal antibody for IAC purification of diniconazole from complex sample matrices was also established. The efficiency of a sol-gel-based IAC method in purifying diniconazole from complex samples was further evaluated by HPLC and ELISA.

**Figure 1:**
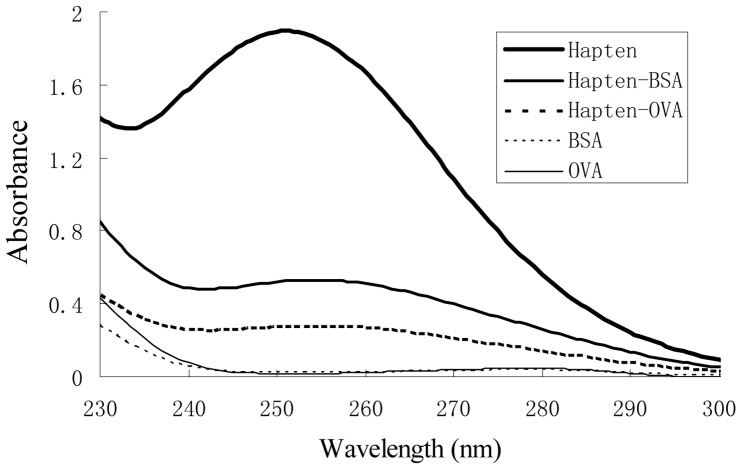
The ultraviolet adsorption spetra of hapten, BSA, OVA and the conjugate. Concentrations of BSA, OVA, and Hapten were 100, 100 and 50 mg L^−1^.

## Materials and Methods

### Instruments and Reagents

Pesticide standards used for cross-reactivity studies were supplied by Jiangsu Qizhou Chemical Group Co., Ltd. (Jiangsu, China). Stock solutions of diniconazole and its analogs (uiconazole, hxaconazole, tbuconazole, tiadimefon, futriafol, eoxiconazole and cproconazole) were prepared in methanol, and stored at 4°C in dark vials. Working standards at various concentrations were prepared from the stock solutions in methanol-phosphate buffer saline solution 20∶80 (v:v) and kept in refrigeration (4°C) when not in use; these standards were daily renewed. Ninety-six-well polystyrene microplates (MaxiSorp) were purchased from Nunc (Roskilde, Denmark). UV spectra were recorded on a DU 800 spectrophotometer (Beckman Coulter, USA). ELISA plates were washed with a Wellwash Plus (Thermo, USA). Absorbances were read with an Infinite M200 microtiter plate reader (Tecan, Switzerland) at 490 nm. Diniconazole was separated using Agilent 7890A GC and Agilent 1200 HPLC (Agilent, USA). Bovine serum albumin (BSA), ovalbumin (OVA), Freund’s complete and incomplete adjuvants, goat anti-mouse IgG-horseradish peroxidase, hydrogen peroxide (H_2_O_2_, 30%), o-phenylenediamine (OPD), tetramethoxysilane (TMOS) and polyoxyethylene sorbitan monolaurate (Tween-20) were purchased from Sigma Chemical Co. (St. Louis, USA). All reagents and solvents were of analytical grade. The BALB/c mice were purchased from the Center of Comparative Medicine of Yangzhou University (Yangzhou, China). All animals used in this study, and animal experiments, were approved by Department of Science and Technology of Jiangsu Province. The license number was SYXK (SU) 2010-0005.

**Figure 2:**
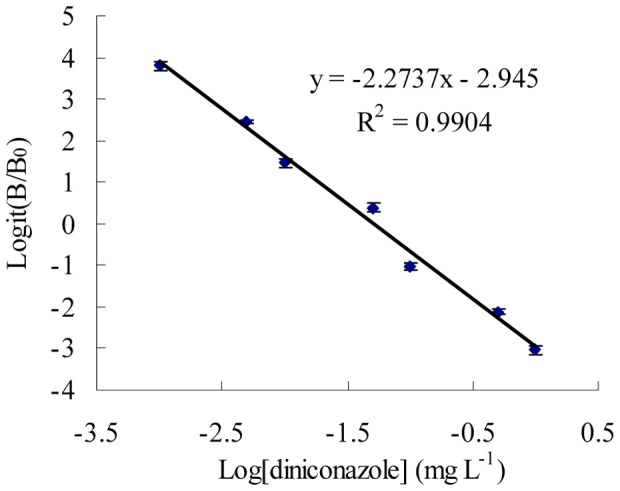
Standard curves by ELISA for diniconazole. ELISA concentrations were the values of three measurements. The error bars indicate standard deviations (n = 3). 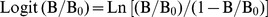.

### Buffers and Solutions

Phosphate-buffered saline (PBS, 0.01 mol L^−1^, pH 7.4), carbonate-buffered saline (CBS, 0.05 mol L^−1^, pH 9.6), and phosphate-buffered saline containing 0.05% Tween-20 (PBST) were used. The substrate solution contained 0.025 mol L^−1^ citrate and 0.062 mol L^−1^ sodium phosphate, pH 5.4. The OPD solution contained 0.4 mg mL^−1^ OPD and 0.012% H_2_O_2_ in the substrate solution.

**Figure 3:**
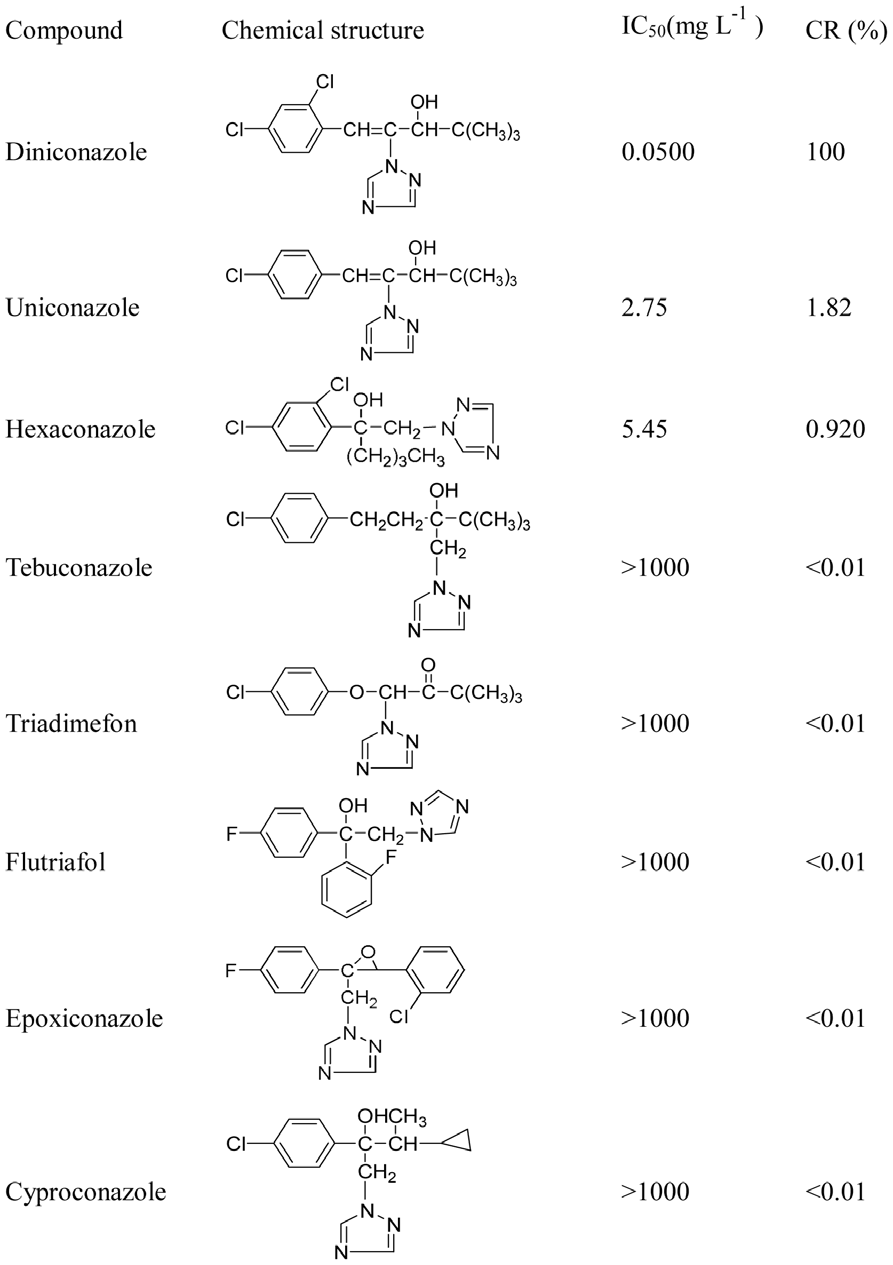
Cross-reactivity of diniconazole and some of its analogs.

### Hapten Synthesis

Diniconazole hapten was synthesized from diniconazole and succinic anhydride in acylation reaction as reported previously [13].

**Figure 4:**
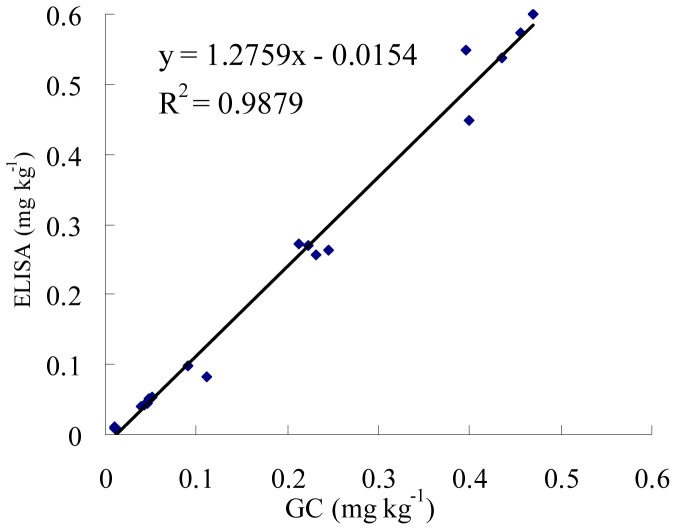
Regression curve of GC versus ELISA methods for determination of diniconazole in water, pear and tomato samples. ELISA concentrations were the mean values of triplicate measurements.

### Immunization and Prepration of Antibodies

Most of pesticides can not induce antibodies directly for they are small molecules (the molecular weight is less than 1000) and lack of T cell epitopes. But after pesticides were conjugated to carrier proteins, T cell epitopes of the conjugation can induce B cells to produce specific antibodies indirectly.

**Figure 5:**
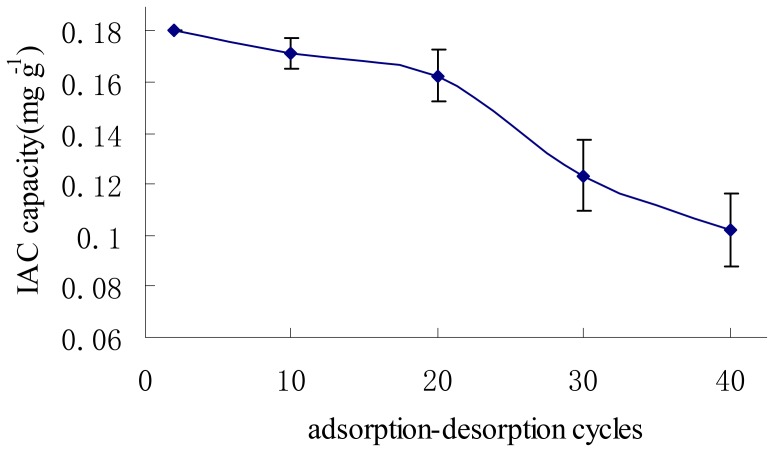
The influence of IAC capacity after reuse. The error bars indicate IAC capacity deviations with three columns.

Diniconazole hapten was coupled to BSA using the active ester method [16] to produce the immunogen and was conjugated with OVA via the mixed anhydride method to produce the coating antigen [17]. The formation of conjugates was confirmed by UV-Vis spectroscopy. Six-week-old female BALB/c mice were immunized with hapten-BSA by intraperitoneal injection according to the methods described by Kishiro et al. [18]. The first injection was of immunogen (100 µg) dissolved in physiological saline and emulsified with an equal volume of Freund’s complete adjuvant. Four subsequent injections were given at 2-week intervals using the immunogen emulsified with Freund’s incomplete adjuvant. A week after the fifth immunization, antiserum was obtained from the tail vein of each mouse. The mice with strong response were subjected to peritoneal cavity injections of 200 µg of immunogen in PBS. Three days after the booster injection, mouse spleen lymphocytes were fused with SP2/0 myeloma cells at a 5∶1 ratio according to the method of Nowinski et al. [19]. The fused cells were cultured with a hypoxanthine- aminopterin-thymidine (HAT) for 2 weeks and in hypoxanthine-thymidine (HT) selection medium for 4 weeks. Culture supernatants were screened for antibody specificity against diniconazole by competitive ELISA, and hybridoma cells in ELISA-positive wells were cloned by the limiting dilution method. Stable antibody- producing clones were expanded. Ascites fluid was obtained from BALB/c mice primed with Freund’s incomplete adjuvant by intraperitoneal (Ip) injection of hybridoma cells. Antibodies were purified using the salting out method with saturated ammonium sulfate [20] and were stored at −20°C after freeze-drying.

**Table 1:** Influence of matrix dilution on the reliability of the diniconazole ELISA.

Spiked level (mg kg^−1^ )	Assay dilution factor^a^	tomato juice		pear juice	
		Mean recovery±SD(%, n = 5)	RSD (%)	Mean recovery±SD(%, n = 5)	RSD (%)
0.5	2.5	118±6.15	5.21	139±4.48	3.22
	5	97.0±4.21	4.34	137±8.51	6.21
	10	96.0±3.24	3.37	117±6.13	5.24
	15	93.6±4.04	4.32	83.8±6.04	7.21
0.1	2.5	330±9.11	2.76	363±13.72	3.78
	5	98.0±6.09	6.21	123±6.90	5.61
	10	89.0±3.17	3.56	100±3.21	3.21
	15	69.0±2.98	4.32	71.0±3.24	4.57
0.05	2.5	358±21.95	6.13	312±19.38	6.21
	5	132±7.05	5.34	88.0±3.84	4.36
	10	100±3.36	3.36	78.0±4.14	5.31
	15	66.0±8.65	13.1	48.0±4.90	10.2
0.01	2.5	224±72.13	32.2	235±73.56	31.3
	5	200±42.40	21.2	152±26.14	17.2
	10	51±8.87	17.4	34±4.86	14.3
	15	32±7.46	23.3	26±4.47	17.2

a tomato and pear samples were diluted (2.5, 5, 10, and 15-fold) with PBS containing 20% methanol.

**Table 2:** Diniconazole recovery from the spiked samples by HPLC and the ELISA.

Sample	Spiked concentration(mg kg^−1^)	HPLC		ELISA	
		Mean recovery±SD(%, n = 5)	RSD(%)	Mean recovery±SD(%, n = 5)	RSD(%)
soil	0.5	91.9±1.55	1.69	90.9±2.76	3.04
	0.1	91.3±0.80	0.876	94.7±4.92	5.20
	0.05	91.8±2.71	2.95	96.1±0.74	0.770
	0.01	90.3±3.21	3.55	93.5±3.23	3.45
wheat flour	0.5	89.0±3.55	3.99	91.4±3.47	3.80
	0.1	92.0±3.50	3.80	93.1±4.31	4.63
	0.05	89.5±2.85	3.18	91.2±4.62	5.07
	0.01	90.5±4.23	4.67	92.4±5.31	5.75
tomato	0.5	91.1±2.27	2.49	89.2±1.71	1.92
	0.1	90.4±2.60	2.88	91.1±5.45	5.98
	0.05	91.3±3.16	3.46	90.0±2.65	2.94
	0.01	91.2±2.67	2.93	91.1±3.42	3.75
apple	0.5	88.5±3.10	3.50	89.3±2.37	2.65
	0.1	92.6±1.42	1.53	91.4±3.17	3.47
	0.05	91.2±1.79	1.96	92.5±3.25	3.51
	0.01	93.4±2.34	2.51	92.3±3.21	3.48
pear	0.5	94.7±1.32	1.39	94.0±3.28	3.49
	0.1	91.6±2.94	3.21	91.3±4.49	4.92
	0.05	91.4±3.13	3.42	93.5±5.71	6.11
	0.01	89.4±4.21	4.71	92.7±3.21	3.46
grape	0.5	87.5±5.17	5.91	89.9±1.72	1.91
	0.1	92.6±4.11	4.44	90.6±3.78	4.17
	0.05	92.9±2.63	2.83	93.2±4.78	5.13
	0.01	89.5±3.23	4.73	92.3±3.43	3.72

**Figure 6:**
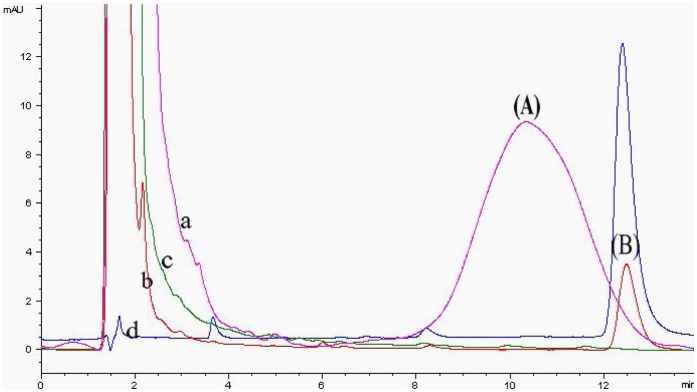
HPLC chromatography of pear samples spiked with 0.1 mg kg^−1^ diniconazole (a: non-purified, b: purified by IAC), blank pear sample (c: purified by IAC) and standard solution of 1 mg kg^−1^ diniconazole (d). Peak identifications: (A). impurity, (B). diniconazole.

### Indirect Competitive ELISA

A checkerboard titration assay was carried out with different amounts of the coating antigen (hapten-OVA) in CBS and of the antibody in PBS [21]. After the screening of the antibody and the coating antigens, an indirect competitive ELISA was developed as follows: the microplates were coated for 2 h at 37°C with 100 µL/well of the coating antigen (0.3 mg L^−1^) in CBS. The plates were washed three times with PBST. Then, 200 µL of PBS containing 1% OVA was added, and the plates were incubated for 30 min at 37°C. Then, the plates were washed again, and 50 µL of sample or standard in PBS containing methanol at different concentrations (10, 20, 30 and 40%, v:v) was added along with 50 µL of 300 mg L^−1^ antibodies in PBS with different pH values (4.5, 5.5, 6.5, 7.5, 8.5 and 9.5) and containing Na^+^ at different concentrations (0.1, 0.2, 0.3, 0.4, 0.5 mol L^−1^). The plates were then incubated for 1 h at 37°C and washed again. Then, 50 µL/well of diluted (1∶20000) goat anti-mouse IgG-horseradish peroxidase was added, and the plates were incubated for 1 h at 37°C. After another washing step, 100 µL/well of the OPD solution was added, and the plates were incubated for 15 min at 37°C. Finally, 2 mol L^−1^ of sulfuric acid (50 µL/well) was added, and the absorbance was measured at 490 nm.

The standard curve for diniconazole was obtained by plotting the Napierian logarithm of the percent binding (logit (B/B_0_)) versus the logarithm of the concentration of diniconazole (logC). The %(B/B_0_) values were calculated using the following equa- tion:
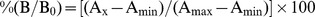
Where Ax is the absorbance of the sample, A_max_ is the absorbance in the absence of analyte, and A_min_ is the absorbance of the background.

The several parameters, including standards in PBS containing methanol concen- trations, antibodies in PBS with different pH values and Na^+^ concentrations, were optimized based on the lowest of the 50% inhibition concentration (IC_50_) and the coefficient of correlation (R^2^) of the linear equation.

### Determination of Cross-reactivity

Under the optimum conditions, cross-reactivity was studied using standard solutions of diniconazole and its analogs.

### Sample Preparation

No specific permits were required for the described field studies. The soil used was collected from the upper 0–20 cm of the horizon in an agricultural field, which is comprised of agricultural land that is owned and maintained by Nanjing Agricultural University specifically for research trials. These field studies did not involve endangered or protected species.

The soil used in the methods development and validation was air dried and sieved through a 4 mm sieve before use. Apple, pear, grape, tomato and wheat flour samples without diniconazole were obtained from a local supermarket.

Spiked samples which were prepared by adding aliquots of diluted standard solutions in methanol were left overnight.

### ELISA Analysis

Distilled water, tap water and pond water samples were spiked with diniconazole standards at 0.01, 0.05, 0.25 and 0.5 mg L^−1^. To reduce the matrix effect, the samples were diluted twice with PBS containing 30% methanol and analyzed by ELISA. Pear and tomato samples (10 g) were ground, spiked with diniconazole standards at 0.05, 0.1, 0.25 and 0.5 mg kg^−1^. The samples were thoroughly mixed, and then allowed to stand at room temperature overnight (12 h). They were mixed with 30 mL methanol, submitted to ultrasonic extraction for 10 min and then centrifuged at 4000×g for 10 min. The 2 mL of supernatant was diluted ten times with PBS and analyzed by ELISA. The recoveries and relative standard deviation (RSD) were calculated.

### Evaluation of the Assay by GC

The above spiked samples were extracted according to the method of Feng et al. [5] and were analyzed by GC-ECD. The measured results were compared with the ELISA results. The GC-ECD analysis was performed on a HP-17 fused silica capillary column (30 m×320 µm×0.25 µm). The GC conditions were 190°C for 0.5 min, a temperature increase to 230°C at rate of 8°C min^−1^ and hold for 6 min; a carrier gas (N_2_) flow rate of 3.0 mL min^−1^; An injection temperature of 250°C using the splitless mode; and a detector temperature of 330°C.

### Preparation of the IAC

The mixture of 1.7 mL of TMOS, 100 µL of methanol, 300 µL of 50% glycerol, 250 µL of distilled water, and 100 µL of 0.04 mol L^−1^ HCl was stirred to obtain a silica sol and then swirled in an ice-bath for 20 min [22], [23]. A 100 µL of 300 mg L^−1^ monoclonal antibody solution and 900 µL of PBS were added to 1 mL of silica sol under stirring. Control columns, which didn’t contain antibody, were prepared in the same manner, except that the 100 µL of 300 mg L^−1^ monoclonal antibody solution was instead of 100 µL of PBS. Other producers of control column were same with IAC column. The gel was weighed and allowed to age at 4°C until a weight loss of 56% original weight was achieved. Afterward the resulting amount of silicate glass (1 g) was ground in a mortar and packed into a 5 mL column. The IAC was subsequently eluted with 10 mL PBS and 10 mL water and stored in PBS at 4°C.

### Extraction with IAC

Soil, apple, pear, grape, tomato and wheat flour samples were spiked with diniconazole at various levels (0.01, 0.05, 0.1 and 0.5 mg kg^−1^). The samples were thoroughly mixed, and then allowed to stand at room temperature overnight (12 h). The soil and wheat flour samples (10 g) were extracted twice by ultrasonic extraction for 15 min with 20 mL acetone and centrifuged at 4000×g for 10 min. The organic phase was collected and evaporated to dryness, and then dissolved in 5 mL of PBST. Fruit samples (10 g) were ground, extracted by shaking for 1 h with 40 mL of acetonitrile. The organic phase was evaporated to dryness and dissolved in 5 mL of PBST.

The extract was passed through the IAC. The column was successively washed with 5 mL PBS, 5 mL water, and 5 mL PBST at a flow rate of 0.5–1 mL min^−1^ in order to remove impurity. Diniconazole was eluted with 1 mL 30% first followed by 2 mL 50% (v:v) methanol in water, and the eluate were mixed.

### Analysis Samples by Both ELISA and HPLC

The above eluate were analyzed by the ELISA (diluted twice) and HPLC [8] using an Eclipse XDB-C_18_ column and ultraviolet photometric detector. The chromatographic isocratic elution was performed (methanol-0.05% H_3_PO_4_ water, 70∶30, v:v) at a flow rate of 1.0 mL min^−1^, and the analytes were detected at the wavelength of 258 nm. The column temperature was 30°C, and the injection volume was 20 µL.

## Results and Discussion

### The Coupled Identification of an Artificial Antigen

UV-Vis spectra showed qualitative differences between the conjugate and the corresponding carrier protein (**Figure 1**). The characteristic absorbance for hapten- BSA showed a blue-shift at 255 nm compared with the 278 nm for BSA, indicating the successful conjugation between hapten and BSA. The coating antigen hapten- OVA gave a UV spectrum similar to that of hapten-BSA. The molar ratios were estimated to be 9∶1 and 4.5∶1 for the immunogen and the coating antigen, respectively.

### Development of the ELISA

Methanol is a common solvent used in immunoassays to improve analyte solubility [20]. The salt concentration, which affected antibody binding [20], and the pH were evaluated to improve the sensitivity of the ELISA. The optimum parameters of the ELISA procedure were 20% methanol, pH 6.5–8.5, and an ionic strength of 0.2 mol L^−1^.

Under the optimum conditions, the ELISA procedures were conducted in triplicate using a series of concentrations of diniconazole. The standard curve was presented in **Figure 2**. It was observed that between the logit (B/B_0_) and logarithm of the concen- tration of diniconazole had good linearity in the range of 0.001 to 1.000 mg L^−1^. The following equation was obtained: logit (B/B_0_) = −2.2737 logC–2.945, R^2^ = 0.9904. An IC_50_ value of 0.050 mg L^−1^ and a limit of detection (LOD, IC_10_) of 5.47 µg L^−1^ were obtained.

### Specificity

The characterization of the monoclonal antibodies indicated a high specificity for diniconazole. There was no obvious cross-reactivity (CR) with most of the triazoles tested; only uniconazole and hexaconazole showed any cross-reactivity (**Figure 3**), the highest cross-reactivity was found to be uniconazole (1.82%), due to the same structure except for the number of chloro substituents on the benzene between uniconazole and diniconazole [13]. The values of cross-reactivity for both dinicona- zole and hexaconazole were 0.920% which may be due to a similar structure for the 2, 4-dichlorophenyl group.

### Correlation between the ELISA and GC

Five types of samples (distilled water, tap water, pond water, pear and tomato) were analyzed by the ELISA and GC. The results were presented in **Figure 4**. A good correlation was obtained between the ELISA (Y) and GC (X) results, with a linear regression equation of Y = 1.2759 X–0.0154 (R^2^ = 0.9879, n = 19). These results suggested that the diniconazole in the samples could be simply, rapidly and accurately detected by ELISA.

### The Mechanism of the IAC Cleanup of Sample Matrix

The IAC techniques take advantages of the high affinity, high specificity and reversible binding characteristics of the antigen-antibody reaction. Sample of diniconazole retained in immunoaffinity column was due to antibody-antigen interact- tions (diniconazole and its antibodies) and not to non-specific adsorption on the solid support. Most of matrix in the sample would not be retained and a small amount of matrix nonspecifically retained, but they usually weakly reserved and can be cleared out of the immunoaffinity column using appropriate eluent.

Organic solvents can affect the action of bio-molecules, modifying their binding thermodynamics; in the same way, the nature of the solvent has an influence on the antibody binding event. Methanol solution was employed for the loading of samples onto the IA columns as well as for the binding and releasing of the target analytes. Under the optimum conditions, diniconazole was eluted with 1 mL 30% first followed by 2 mL 50% (v:v) methanol in water. The antigen-antibody complex was dissociated in the methanol modified environment of the immunoaffinity column. After each adsorption-desorption cycle, the immunoaffinity column must be left in PBS and water to regenerate the antibody before the next use.

### Characterization of the IAC

The conditions of the IAC were optimized. PBS and water were used as the equilibrium and absorbent media, respectively, and a methanol solution was used as the eluent. **Figure 5** showed the column capacity was 0.180 mg g^−1^, and the capacity gradually reduced to 90.3% of the original column capacity after reuse 20 times in spiked samples.

### Matrix Interference and Recovery Study

Matrix interference is one of the most common challenges in performing immuno- assays on complex samples. Sample dilution was the easiest and most immediate way to minimize matrix effects. To evaluate the influence of the matrix on the immuno- assay, pear and tomato samples were diluted (2.5, 5, 10, and 15-fold) with PBS containing 20% methanol. The results presented in **table 1**, the matrix effects of both samples were reduced to acceptable levels when the samples were diluted 10-fold. The recoveries ranged from 79.2% to 111%, and the RSDs were between 2.16% and 10.3% for tomato and pear. Excessive dilution resulted in an analytical concentration below the LOD (**Table 1**).

In order to achieve improved sensitivity, a reduction in the dilution factor is required. The IAC was used to purify various samples (soil, apple, pear, grape, tomato and wheat flour) containing diniconazole. The results in **Figure 6** and **Table 2** clearly demonstrated the excellent sample purification was achieved with the IAC which allowed precise and accurate determination of diniconazole in complex matrices. In addition, the results (**Table 2**) of the ELISA correlated well with the HPLC results. The average recoveries of diniconazole by HPLC from all samples ranged from 87.5% to 94.7%, which were similar to those of the ELISA.

The IAC and HPLC technique is a basis that coupled column chromatography using on-line sample cleanup with a “tailor-made” copper phthalocyanine-modified silica pre-column is a well-established technique for HPLC analysis of diniconazole in agricultural samples [24].

### Conclusion

An indirect competitive ELISA for diniconazole was developed and was demon- strated to successfully detect diniconazole in water, pear and tomato samples. The ELISA was shown to have a higher sensitivity and specific than those of already reported polyclonal antibody-based ELISA [13], and the sensitivity (IC_50_ value) was 0.0500 mg L^−1^ and the cross-reactivity was below 1.82%. A good correlation between the ELISA and GC results was obtained for the spiked samples. In order to achieve improved assay sensitivity, the IAC was used to remove the interfering components while maintaining good recovery. The accuracy and precision were well within the requirements for residue analysis. Pretreatment reduced solvents and a multi-step extraction procedure.

The IAC and HPLC method was able to eliminate the interference of sample matrices originated from agricultural samples more successfully than the routine detection of diniconazole [10] in respect of the simplicity and the rapidity of the procedure.

Although in this study, we developed an IAC clean-up column for a single pesticide, by using several different antibodies or antibodies with a broad cross-reactivity to pesticides with similar structures, we will develop a multiple analysis for residual pesticides using a tandem IAC clean-up column. The IAC extraction procedure coupled with HPLC and ELISA analysis will provide multi-residue detection methods.

Together, the sol-gel IAC in combination with HPLC and ELISA may provide two alternative approaches for the quantitative and sensitivity analysis of diniconazole levels in environmental and agricultural samples.
